# Supplementary material for: Predisposition footprints in the somatic genome of Wilms tumours
Source: Cancer Discov. Author manuscript; Available in PMC 2025 Jan 9. (PMC7617291; doi:10.1158/2159-8290.CD-24-0878)
Supplement: Supplementary Figures [file EMS202070-supplement-Supplementary_Figures.pdf]

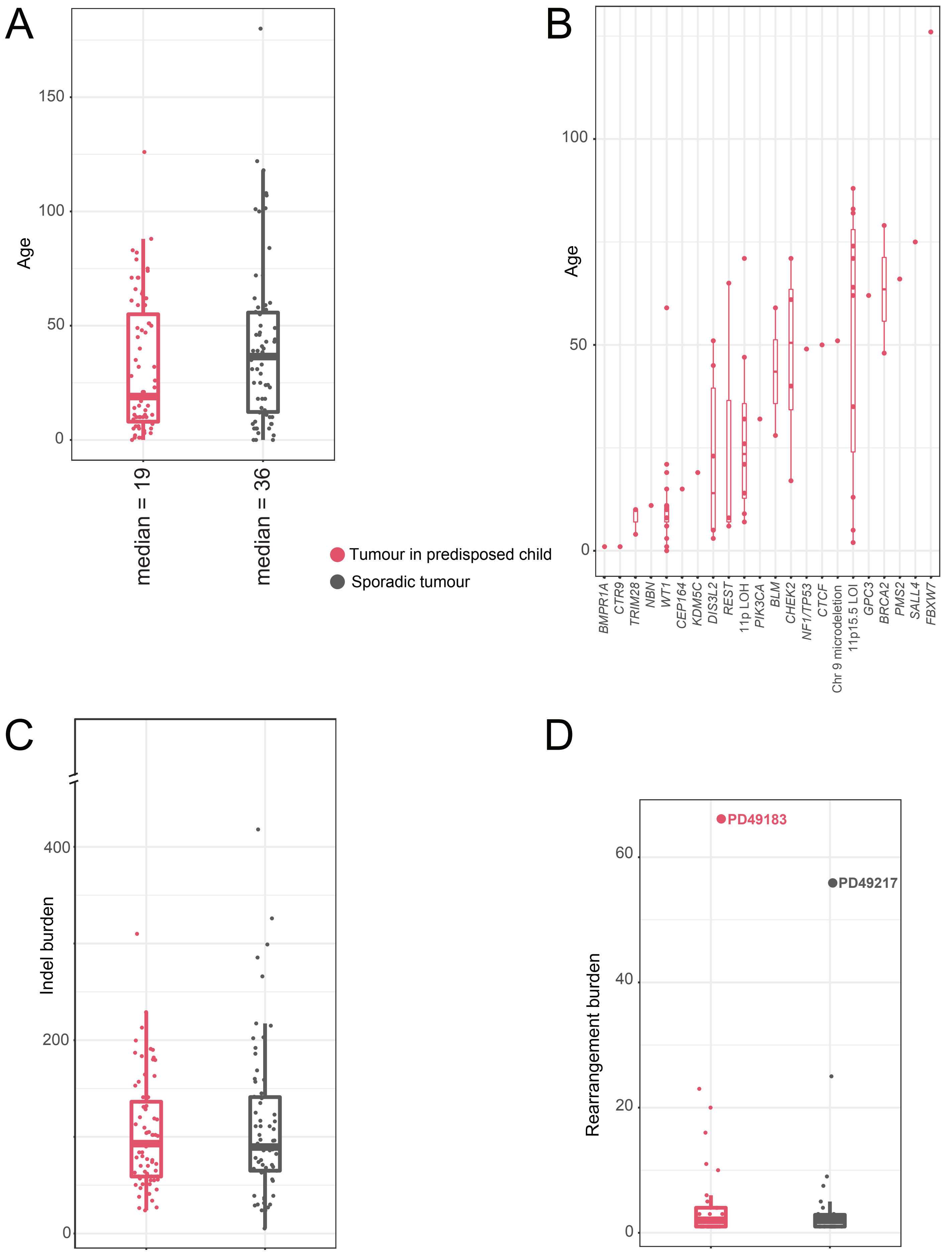

Supplementary Figure 1

A Age at diagnosis (months) in predisposed children versus children with sporadic tumours.

B Age at diagnosis (months) in predisposed children, as per predisposition.

C Indel burden between tumours in predisposed children versus those in sporadic tumours. PD50667 has promoter hypermethylation of *MLH1*.

D Rearrangement burden between tumours in predisposed children versus those in sporadic tumours. PD49183 and PD49217 have mutations in *TP53*.

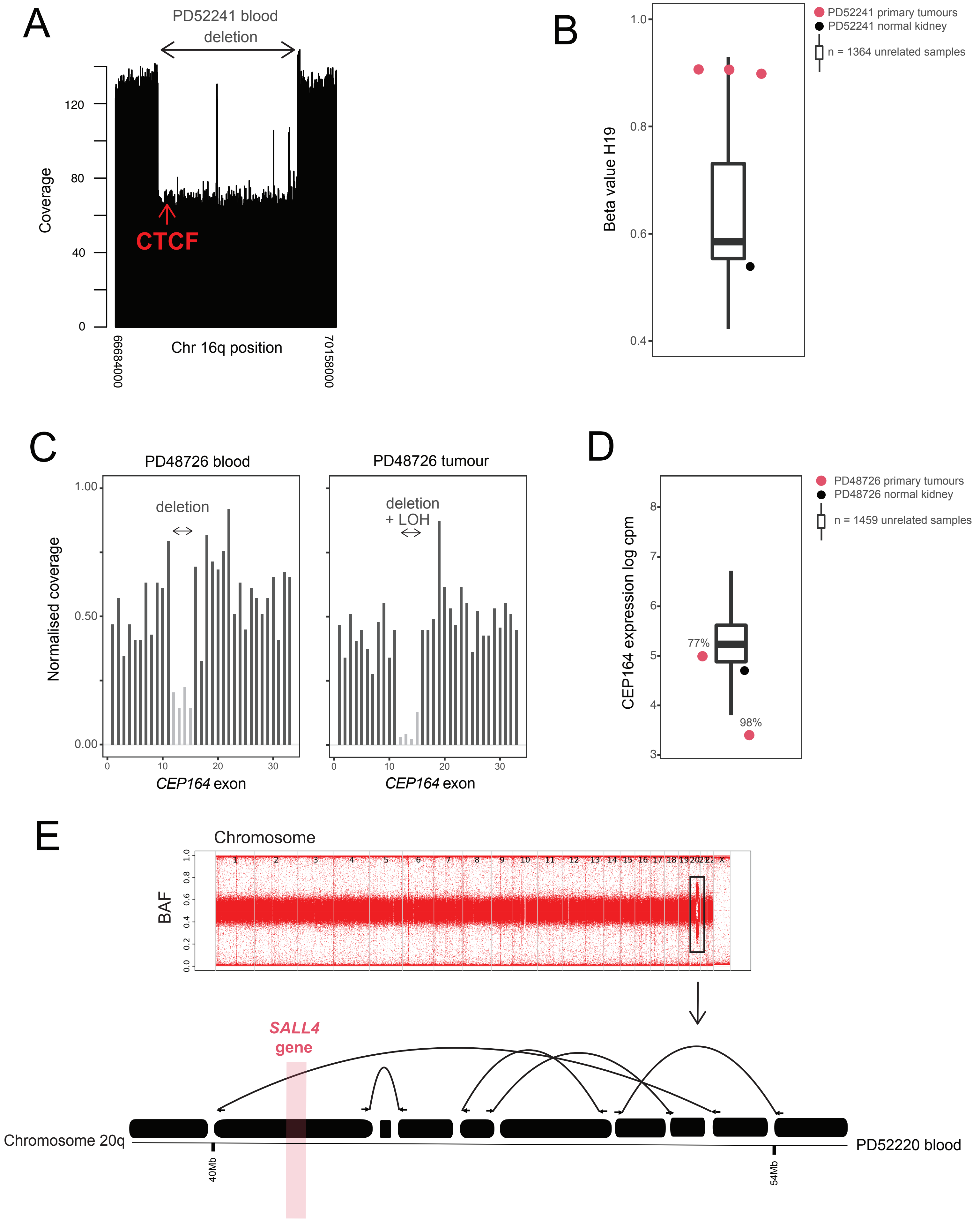

Supplementary Figure 2
A Raw coverage from WGS data showing deletion on chromosome 16q, overlapping *CTCF*, in a blood sample.
B Beta values of H19 from tumours and normal kidney from a child with germline chromosome 16q deletion, compared to 1364 unrelated samples.
C Coverage per exon across *CEP164* in blood and tumour.
D Expression of *CEP164* in tumours and normal kidney from a child with germline *CEP164* deletion, compared to 1459 unrelated samples, numbers show tumour purity.
E Complex germline rearrangement on chromosome 20q, with intact *SALL4*.

A

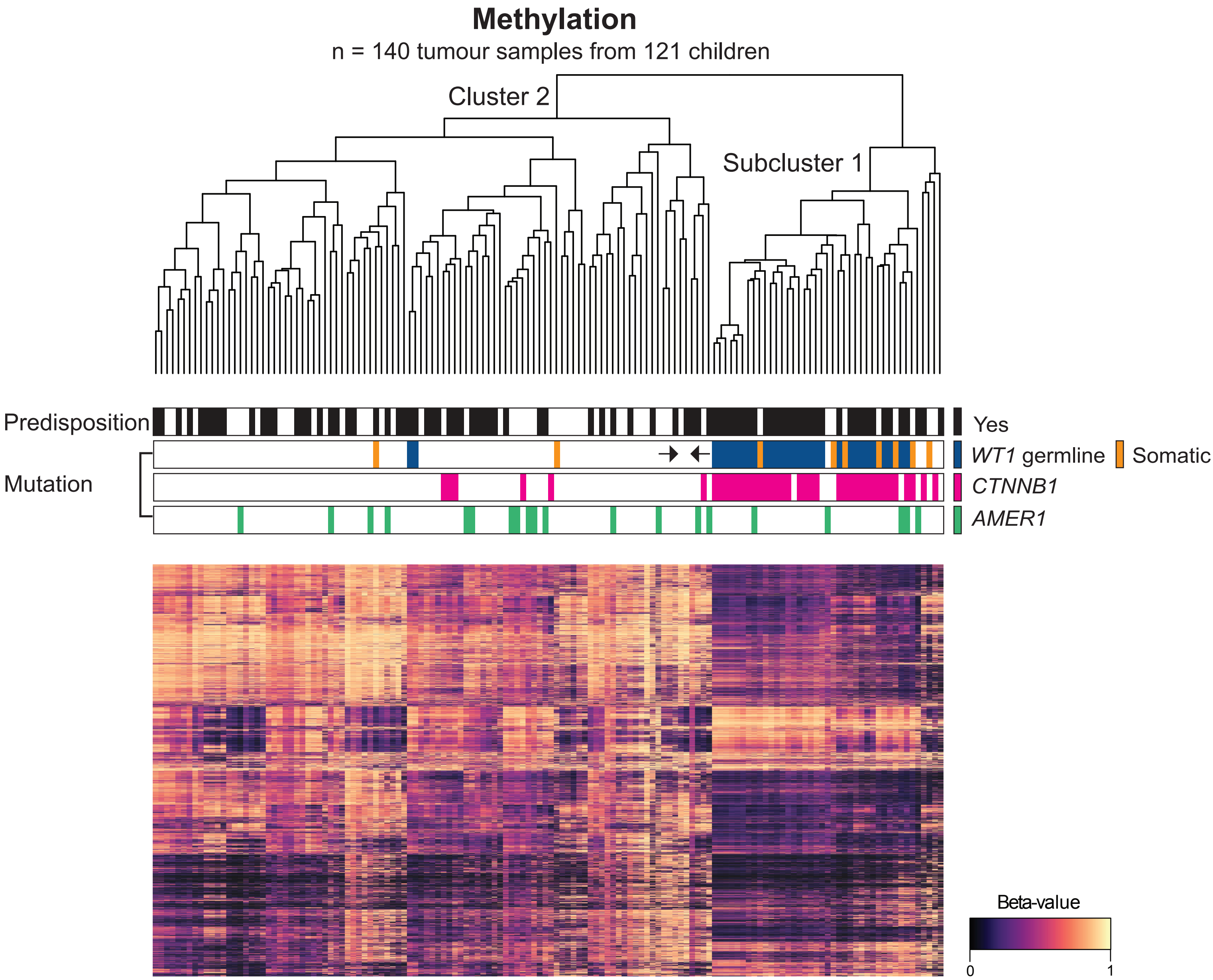

Supplementary Figure 3

A Global methylation pattern of 10,000 most variable probes does not segregate tumours from children with a predisposition versus tumours occurring in children in whom no predisposition could be identified. However, tumours driven by Wnt signalling on a background of germline/mosaic *WT1* or somatic *WT1* cluster separately (p-value $<10^{-27}$, Fisher's exact test). Tumours in Subcluster 1 that lack *WT1* and *CTNNB1/AMER1/PIK3CA* are PD50699a, PD50591c, PD50675a and PD49189c. Arrows indicate nephrogenic rests (PD50643l and PD50602d).

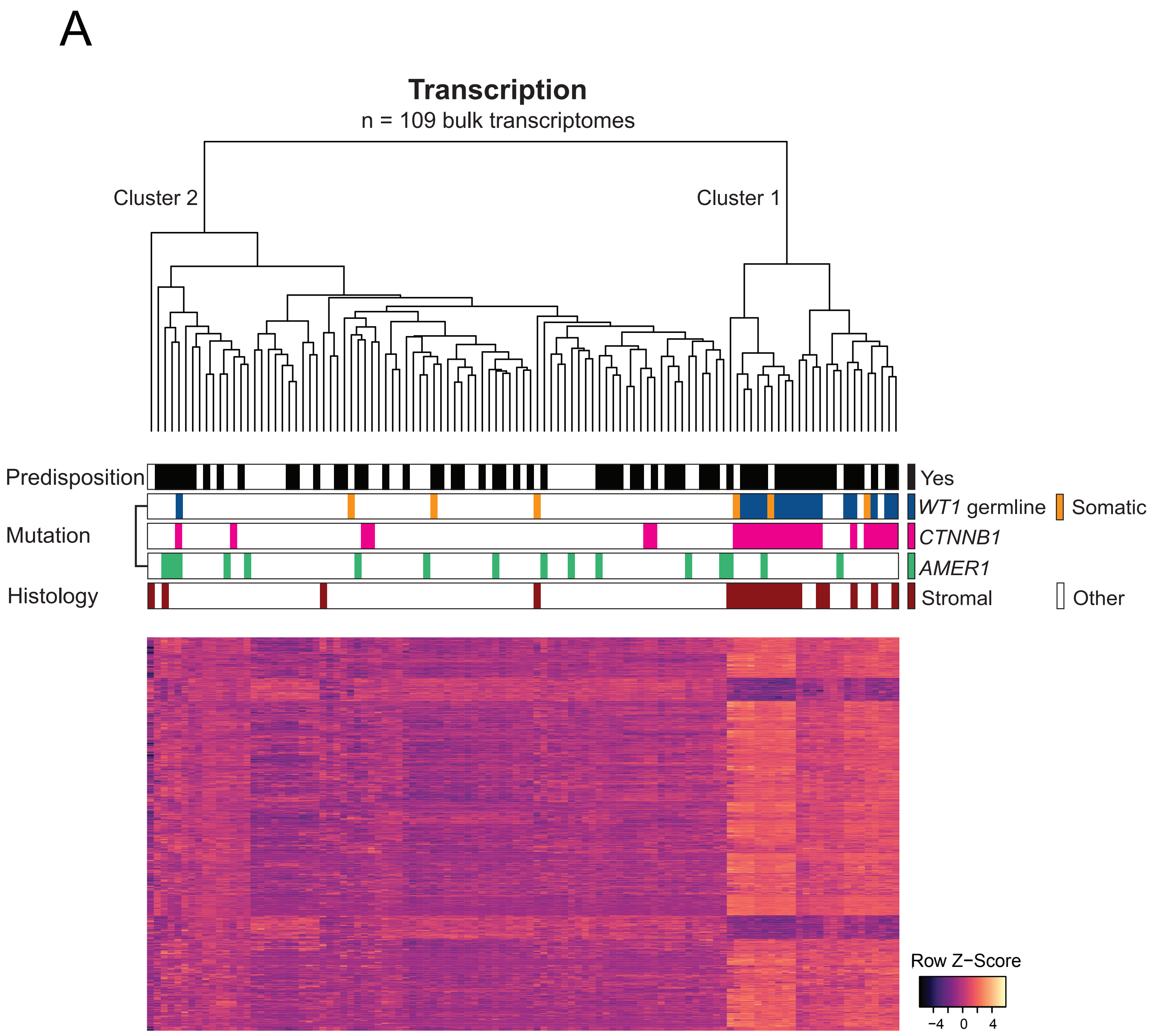

Supplementary Figure 4

A Differential gene expression analysis does not segregate tumours from children with a predisposition versus tumours occurring in children in whom no predisposition could be identified. However, there are 842 differentially expressed genes (with a log fold change greater than 1) between tumours driven by Wnt signalling on a background of germline/mosaic/somatic *WT1* and the remainder of the cohort. This group of tumours cluster in Cluster 1 (p-value < 10^{-14}, Fisher's exact test). Germline *WT1* sample in Cluster 2 is from PD50733 (a child with WAGR syndrome). Tumours in Cluster 1 that lack *WT1* and mutant *CTNNB1/AMER1/PIK3CA* are PD48715a, PD48719a, PD50593a, PD50719a, PD50665a and PD52202c. Stromal histology is highlighted to demonstrate that clustering is not due to histology alone.

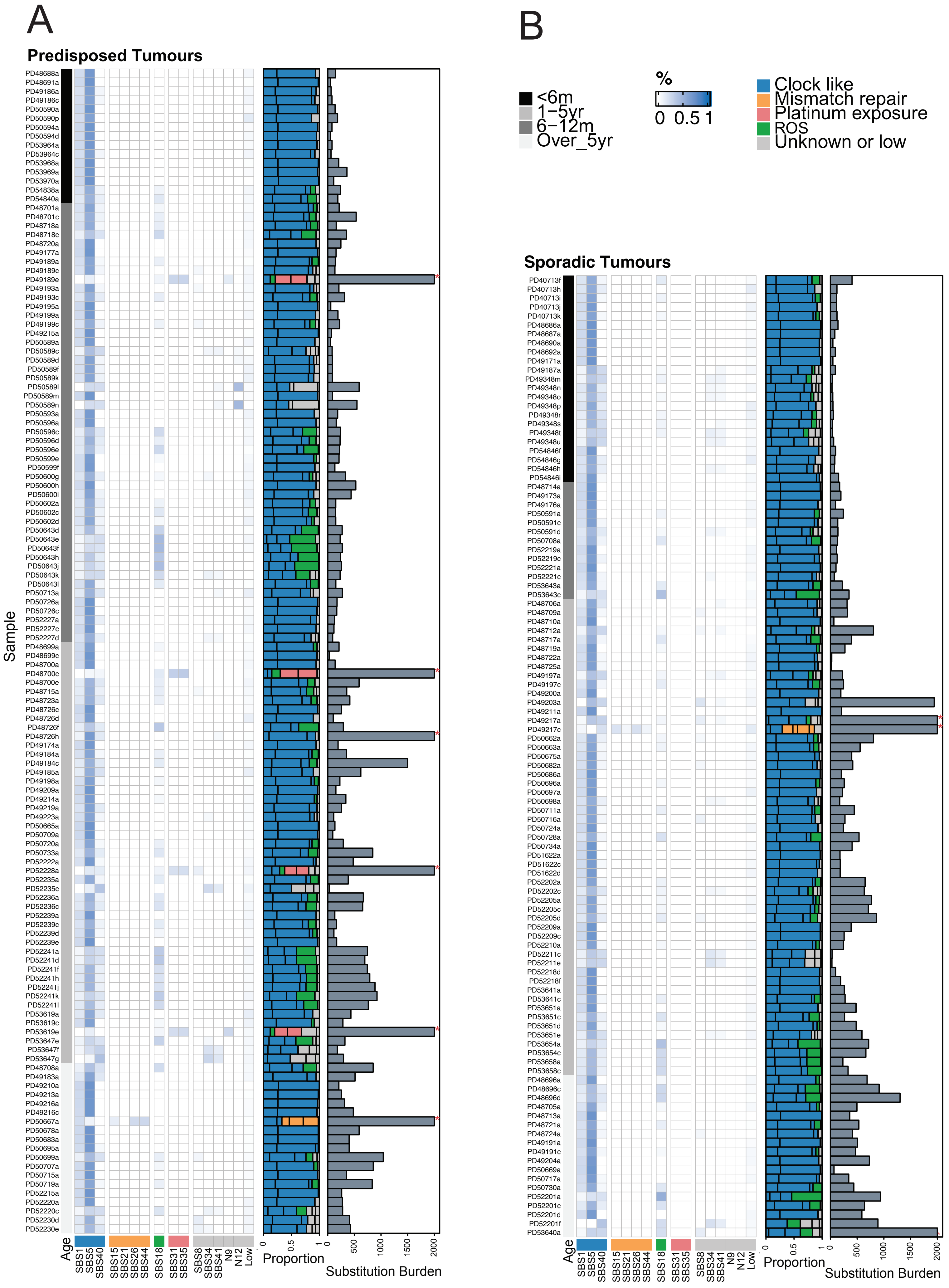

Supplementary Figure 5

A Mutational signatures in tumours from predisposed children.

B Mutational signatures in sporadic tumours.

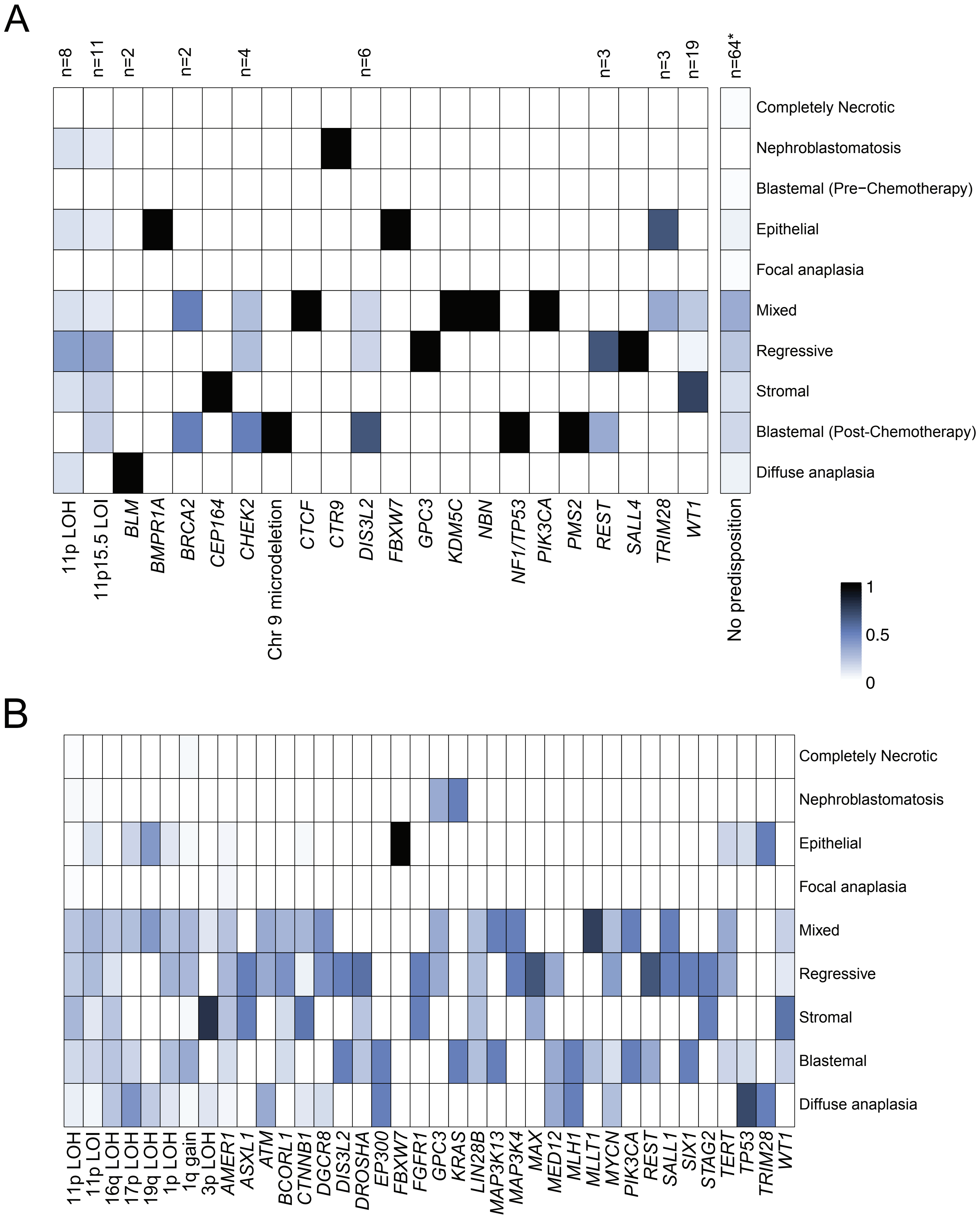

Supplementary Figure 6

A Histological subtype amongst predispositions and amongst cases without a predisposition. Colour refers to percentages of cases within each group with each histology. For predispositions with more than one case, number of cases is shown by n. *Histological information was not available for 2 cases.

B Histological subtype amongst somatic drivers. Colour refers to percentages of cases within each group with each histology. Drivers are only included if occurring in more than one case.

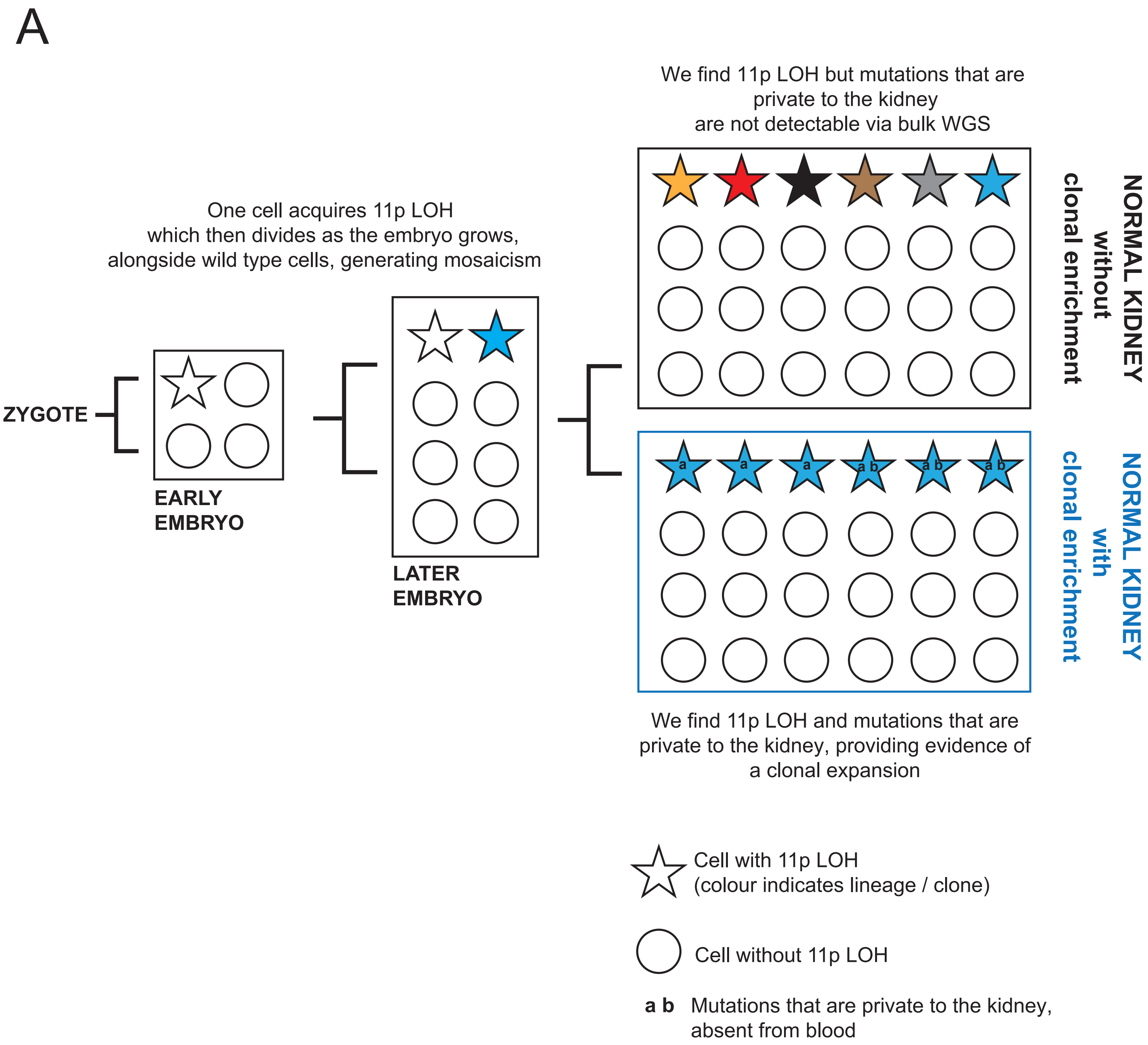

Supplementary Figure 7

A Polyclonal versus clonal enrichment of 11p loss of heterozygosity (LOH) in normal kidneys.

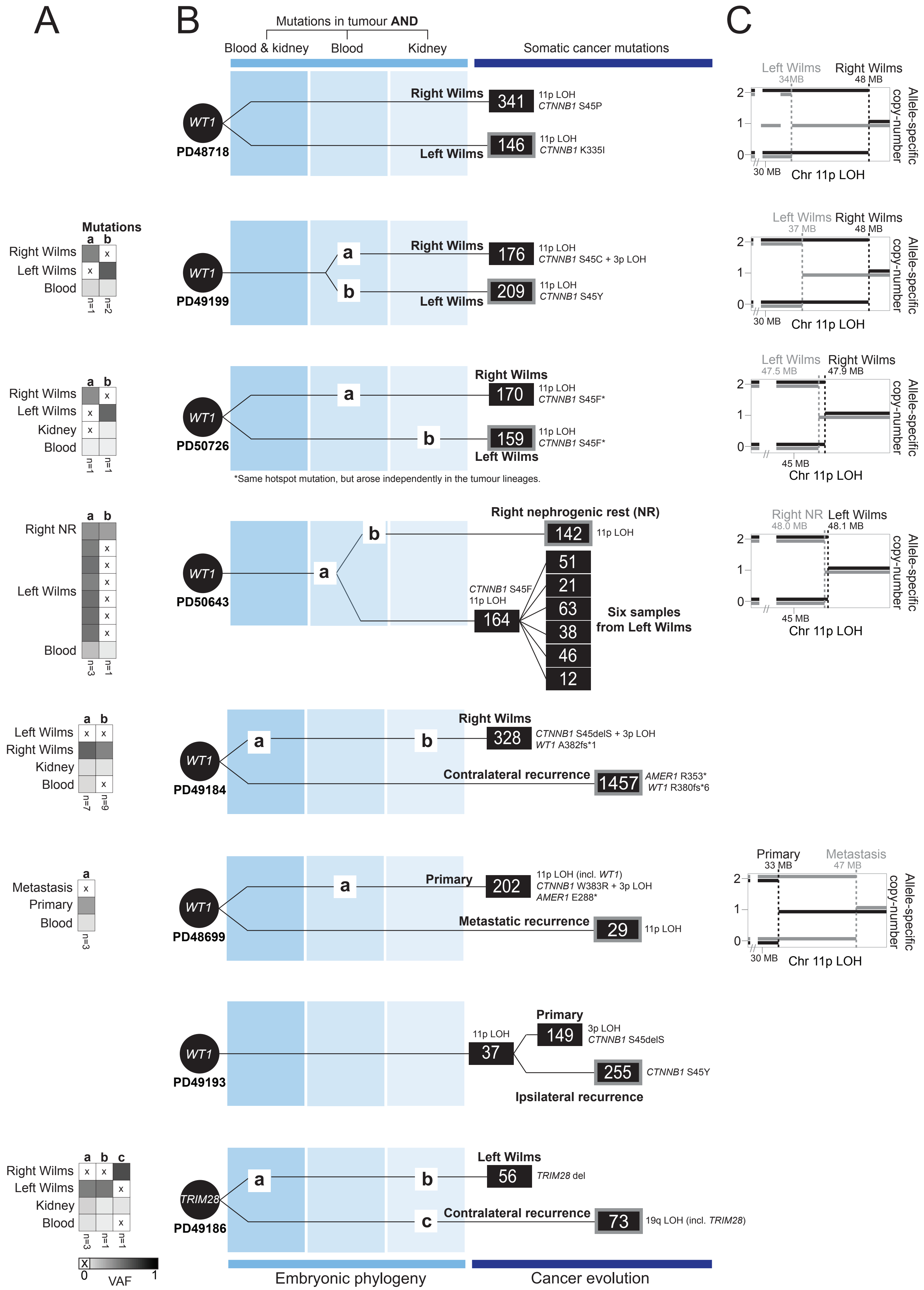

Supplementary Figure 8 Phylogenies in children with multiple neoplasms and *WT1/TRIM28* predispositions

A Variant allele Frequency (VAF) heatmap of embryonic mutations across all samples from an individual, underlying phylogeny reconstruction.

B Example phylogenies, circle represents zygote with predisposition mutation. Patient ID underneath. Lines (not scaled to mutation burden) represent phylogenetic relations. Number in squares are the tumour substitution burden. Variants listed are the driver events of each neoplasm.

C Noteworthy genomic features showing breakpoints of 11p LOH.

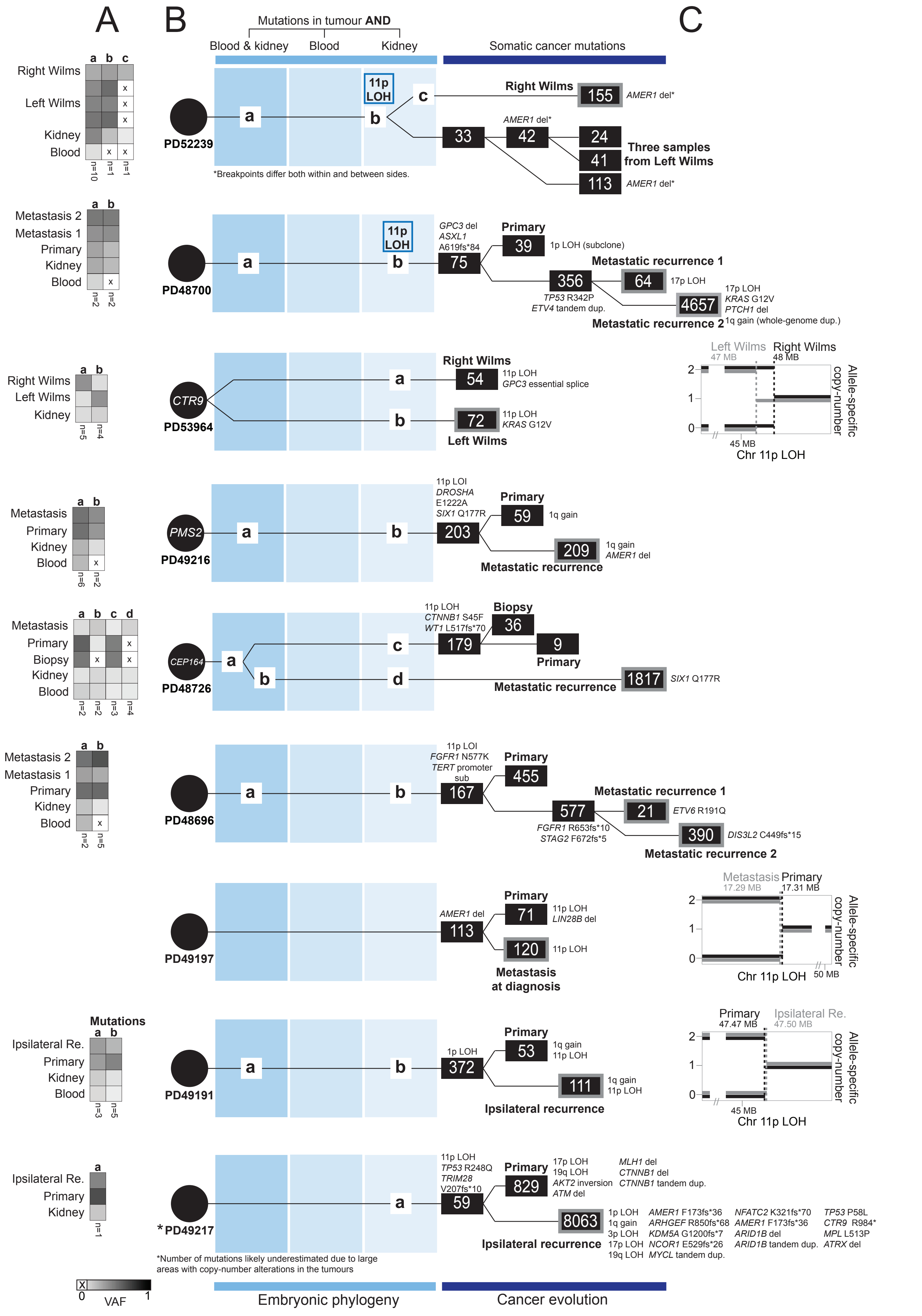

Supplementary Figure 9 Phylogenies in children with multiple neoplasms and either mosaic predispositions, non *WT1/TRIM28* germline predispositions or sporadic tumours

A Variant allele Frequency (VAF) heatmap of embryonic mutations across all samples from an individual, underlying phylogeny reconstruction.

B Example phylogenies, circle represents zygote with predisposition mutation. Patient ID underneath. Lines (not scaled to mutation burden) represent phylogenetic relations. Number in squares are the tumour substitution burden. Variants listed are the driver events of each neoplasm.

C Noteworthy genomic features showing breakpoints of 11p LOH.
